# SET antagonist enhances the chemosensitivity of non-small cell lung cancer cells by reactivating protein phosphatase 2A

**DOI:** 10.18632/oncotarget.6313

**Published:** 2015-11-13

**Authors:** Man-Hsin Hung, Cheng-Yi Wang, Yen-Lin Chen, Pei-Yi Chu, Yung-Jen Hsiao, Wei-Tien Tai, Ting-Ting Chao, Hui-Chuan Yu, Chung-Wai Shiau, Kuen-Feng Chen

**Affiliations:** ^1^ Department of Medical Research, National Taiwan University Hospital, Zhongzheng District, Taiwan; ^2^ National Center of Excellence for Clinical Trial and Research, National Taiwan University Hospital, Zhongzheng District, Taiwan; ^3^ Division of Medical Oncology, Department of Oncology, Taipei Veterans General Hospital, Taipei City, Taiwan; ^4^ Division of Hematology and Oncology, Department of Medicine, Taipei Veterans General Hospital, Taipei City, Taiwan; ^5^ Program in Molecular Medicine, School of Life Science, National Yang-Ming University, Taipei City, Taiwan; ^6^ School of Medicine, National Yang-Ming University, Taipei City, Taiwan; ^7^ Institute of Biopharmaceutical Sciences, National Yang-Ming University, Taipei City, Taiwan; ^8^ Medical Research Center, Cardinal Tien Hospital, Fu Jen Catholic University, New Taipei, Taiwan; ^9^ Department of Pathology, Cardinal Tien Hospital, Fu Jen Catholic University, New Taipei, Taiwan; ^10^ Department of Pathology, Show Chwan Memorial Hospital, Changhua City, Taiwan

**Keywords:** SET, protein phosphatase 2A, non-small cell lung cancer, chemoresistance, Akt

## Abstract

SET is known as a potent PP2A inhibitor, however, its oncogenic role including its tumorigenic potential and involvement in the development of chemoresistance in non-small cell lung cancer (NSCLC) has not yet been fully discussed. In present study, we investigated the oncogenic role of SET by SET-knockdown and showed that SET silencing impaired cell growth rate, colony formation and tumor sphere formation in A549 cells. Notably, silencing SET enhanced the pro-apoptotic effects of paclitaxel, while ectopic expression of SET diminished the sensitivity of NSCLC cells to paclitaxel. Since the SET protein was shown to affect chemosensitivity, we next examined whether combining a novel SET antagonist, EMQA, sensitized NSCLC cells to paclitaxel. Both the *in vitro* and *in vivo* experiments suggested that EMQA and paclitaxel combination treatment was synergistic. Importantly, we found that downregulating p-Akt by inhibiting SET-mediated protein phosphatase 2A (PP2A) inactivation determined the pro-apoptotic effects of EMQA and paclitaxel combination treatment. To dissect the critical site for EMQA functioning, we generated several truncated SET proteins. By analysis of the effects of EMQA on the binding affinities of different truncated SET proteins to PP2A-catalytic subunits, we revealed that the 227–277 amino-acid sequence is critical for EMQA-induced SET inhibition. Our findings demonstrate the critical role of SET in NSCLC, particularly in the development of chemoresistance. The synergistic effects of paclitaxel and the SET antagonist shown in current study encourage further validation of the clinical potential of this combination.

## INTRODUCTION

Non-small cell lung cancer (NSCLC) represents nearly 85% of lung cancer patients and is the leading cause of cancer-related death worldwide [1]. For the majority of patients without particular actionable molecular alterations, namely mutations of epidermal growth factor receptor and fusion mutation involving the anaplastic lymphoma kinase, and those who have progressed on target agents, chemotherapy is the major treatment choice [2–6]. However, the clinical benefit of chemotherapy is largely limited by the occurrence of resistance. Nearly 25% of patients with advanced NSCLC show primary resistance to first-line chemotherapy, and in almost every case eventually leads to on chemotherapy progression [7, 8]. Therefore, it is necessary to understand the possible mechanisms associated with chemoresistance and find effective strategies to improve the chemo-sensitivity of patients.

The SET protein is a potent inhibitor of protein phosphatase 2A (PP2A) [9]. The expression of SET has been found in tumor tissues of different human malignant diseases, such as leukemia, Wilms' tumor, aveolar soft part sarcoma, cancer of colon, breast, and NSCLC [10–16]. It is worth noting that the expression level of SET is closely correlated with cell growth rate; in quiescent or contact-inhibited cells, SET expression is low, but it is significantly elevated in rapidly dividing cells [10]. Importantly, SET promotes tumourigenesis through interacting with several PP2A-regulated oncogenic pathways, including Akt, MAPK, c-JUN, and BCR/ABL [17–22]. Moreover, Cristóbal et al. showed that SET determined the sensitivity to oxaliplatin in colonrectal cancer cells [15]. Overexpression of SET in three different colon cancer cell lines, SW480, HT-29 and LS513, resulted in increasing resistance to oxaliplatin and 5-FU, and silencing SET via siRNA oppositely enhanced their sensitivity to this chemoregimen. Collectively, these data illustrate the putative role of SET in tumourigenesis and drug resistance in human malignant diseases.

Intriguingly, there is a growing body of literature demonstrating the anti-tumor effects of antagonizing SET-mediated PP2A inhibition [11, 14, 16, 23–26]. FTY720, a sphingosine analogue, was found to reactivate PP2A and promote apoptosis of cancer cells by disrupting SET-PP2Ac binding in some malignant diseases, including NSCLC [11, 15, 16, 27]. Furthermore, Bueno and his colleagues demonstrated that treatment with FTY720 for 30-days impaired urethane-induced lung tumor growth in BALB/c mice. [28] These findings suggest the therapeutic potential of applying a SET antagonist for lung cancer treatment. But the exact role of SET in tumourigenesis and chemoresistance of lung cancer has yet not been full characterized.

In the current report, we investigated the relevance of SET in lung cancer and showed that SET was expressed in both lung cancer cell lines and clinical tumor samples obtained from patients with NSCLCs. Furthermore, we observed that SET promotes cell growth and tumor formation in NSCLC cells. Moreover, we found that ectopic expression of SET induces resistance in NSCLC cells to paclitaxel, and, importantly, antagonizing SET via siRNA or a novel SET inhibitor, EMQA, significantly enhances the *in vitro* and *in vivo* anti-tumor effects of paclitaxel.

## RESULTS

### The SET oncoprotein affects cell growth and sphere formation in NSCLC cells

**Figure 1:**
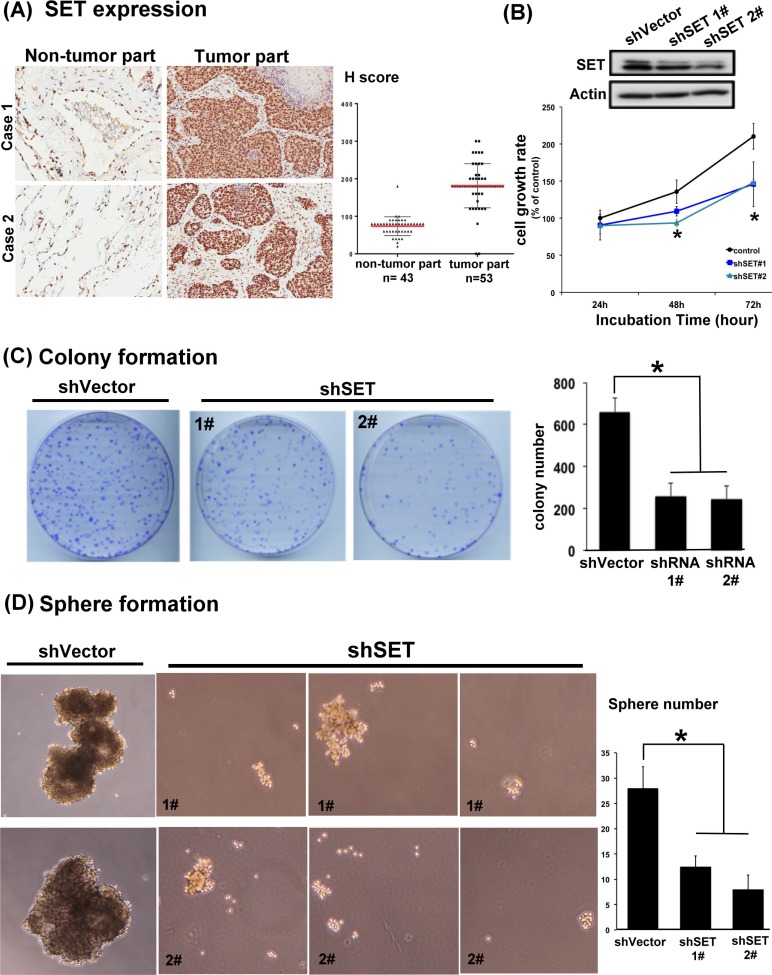
SET is highly expressed in lung tumors and is critically associated with the oncogenic potency of NSCLC cells (**A**) Representative images of the immunohistochemical staining of SET in the normal parts (left column) and tumor parts (middle column) obtained from patients with NSCLCs. H score of each samples analyzed were presented in the dot plot (right column). Red bar: mean, block bar: S.D. (**B**) The average proliferation rate of A549 cells with and without knockdown of SET detected by MTT. (*n* = 6) (**C**) Representative images and quantification of the mean number per dish of the colony formation of A549 cells with and without knockdown of SET. (*n* = 3) (**D**) Representative image and quantification of the mean number per dish of the sphere formation of A549 cells with and without knockdown of SET. (*n* = 3).

To confirm the clinical relevance of SET protein in NSLCL, we first analyzed the presence of SET in the tumor tissue obtained from 53 patients with NSCLC and the adjacent normal parts of lung in 43 patients of this cohort (Table 1 and Figure 1A). Analyzed by immunohistochemical (IHC) stain, 51 patients (96.2%) had SET expression in their tumors. Importantly, the strength of SET expression in tumors was significantly higher than that in the normal tissues; the average H score was 181 in tumor parts and 73.7 in normal parts (Figure 1A). More importantly, we found that high SET expression in tumor part was significantly associated with poor tumor differentiation (*p* = 0.030) and advanced clinical stage of patient (*p* = 0.031, Table 2). To disclose the role of SET in promoting carcinogenesis of NSCLC cells, shRNA against SET was used to knockdown SET in A549 cells. The growth rates and tumourigenecity abilities of these wild-type (WT) and SET-knockdown (SET-KD) A549 cells were assessed by MTT, colony formation and sphere formation assay. As shown in Figure 1B and 1C, genetic knockdown of SET significantly affected the growth rates of A549 cells. The cell growth rate of SET-KD A549 cells determined by MTT was significantly slower than WT cells, and the number of tumor colonies formed at 14 days was significantly reduced in the SET-KD cells, too. The ability of tumor sphere formation was also significantly diminished in these SET-KD cells. (Figure 1D)

**Table 1:** General characteristics of lung cancer cohort (*N* = 53)

Characteristics	Number	%
**Male sex**	**20**	**37.7**
**Median age (IQR)**	**69 (61–79)**	
**Tumor differentiation** **1** **2** **3**	**5** **33** **15**	**9.4** **62.3** **28.3**
**Initial AJCC stage** **1** **2** **3** **4**	**25** **17** **10** **1**	**47.2** **32.1** **18.9** **1.9**
**High SET expression**	18	34
**High p-Akt expression**	11	20.8

IQR, interquartile range; AJCC, American Joint Committee of Cancer

**Table 2:** Characteristics of patients with high and low SET expression

Characteristics	High SET N (%)	Low SET N (%)	*p*
Male sex	5 (27.8)	15 (42.9)	0.221
Median age	69	68	0.777
Poor tumor differentiation	9 (50.0)	6 (17.1)	0.030
Advanced clinical stage	7 (38.9)	4 (11.4)	0.031

### The presence of SET inhibits the activity of PP2A of NSCLC cell and impairs its sensitivity to chemotherapy

**Figure 2:**
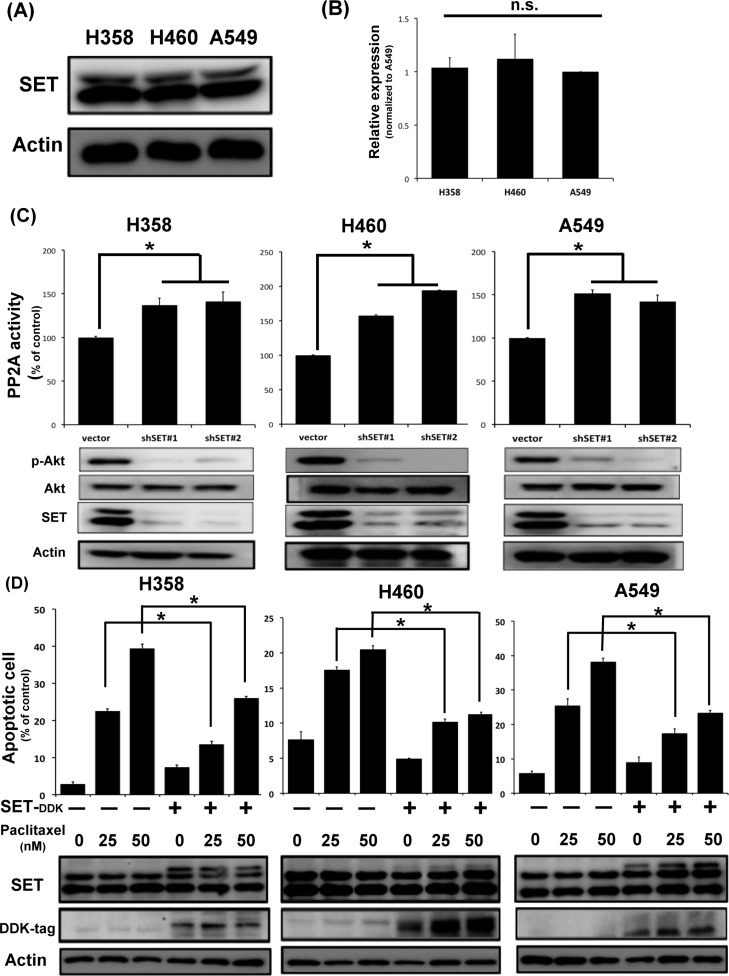
SET expression affects PP2A-mediated p-Ake downregulation and the chemosensitivity of NSCLC cells (**A**) Representative western blot image of the endogenous SET protein expressions in three NSCLC cell lines. (**B**) Relative mRNA expression level of SET in three NSCLC cell lines. (*n* = 3) (**C**) SET knockdown resulted in increasing PP2A activity and decreasing p-Akt expression of NSCLC cells. Upper panel shows results of PP2A activity analysis. Bar: mean, error bar: S.D. (*n* = 3) Lower panel shows representative image of western blot analysis of NSCLC cells with and without SET knockdown. (**D**) Ectopic expression of SET decreased paclitaxel-induced cell death. The percentage of apoptotic cells with or without ectopic expression of SET after exposure to paclitaxel at indicated doses for 48 hours was determined by sub-G1 analysis (upper panel). Bar: mean, error bar: S.D. (*n* = 3).

To understand the biological effects of SET in NSCLC, we first examined the endogenous expression of SET in three different NSCLC cell lines, namely H358, H460 and A549 cells. As shown in Figure 2A and 2B, the three NSCLC cell lines expressed SET similarly in both protein and mRNA level. Furthermore, transient knockdown of SET leaded to increasing PP2A activity and downregulation of p-Akt, one of the major PP2A-regulated oncogenic signals, in all NSCLC cells (Figure 2C). Since chemotherapy is one of the major treatments for NSCLC patients in a setting of almost certain eventual chemoresistance, we next investigated whether SET overexpression affects the sensitivity of lung cancer cells to chemotherapy. Interestingly, we found that the pro-apoptotic effects of paclitaxel were significantly diminished in cancer cells with SET overexpression (Figure 2D). Collectively, above data obtained from NSCLC cell lines and the clinicopathologic analysis of NSCLC patients suggested that SET plays a critical role in promoting carcinogenesis and chemoresistance of NSCLC.

### Antagonizing SET-mediated PP2A inactivation is a feasible approach against NSCLC

**Figure 3:**
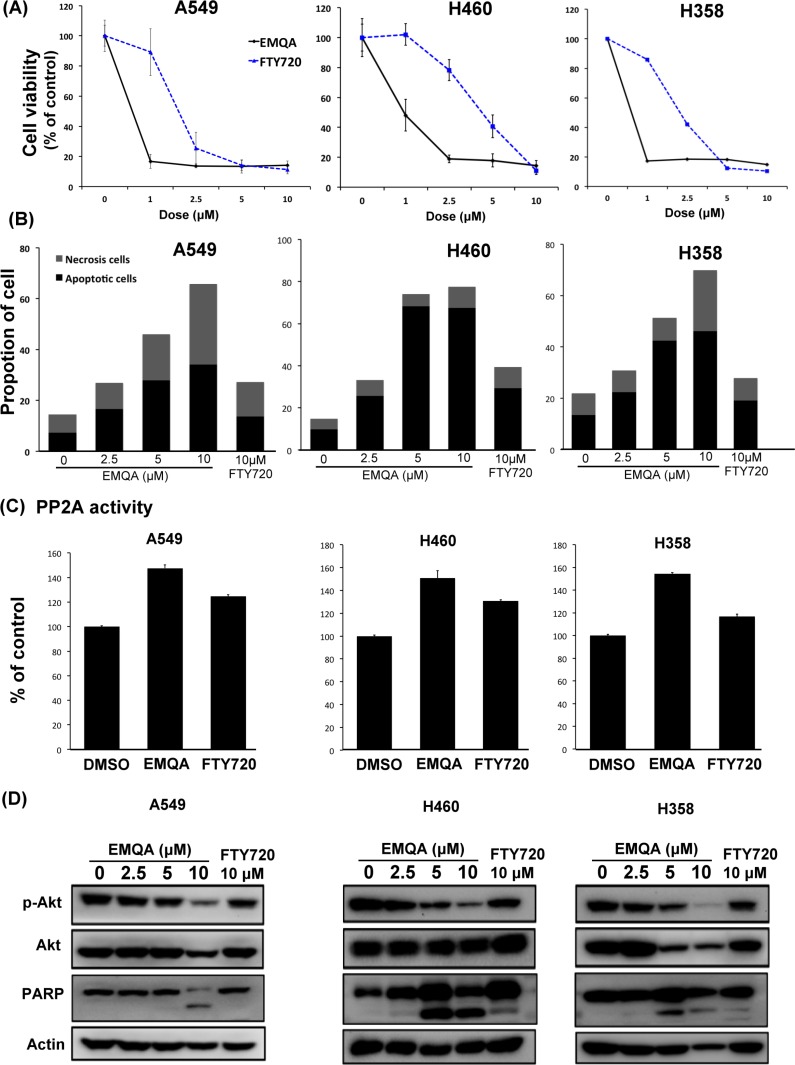
Efficacy of SET antagonist, FTY720 and EMQA, for the treatment of NSCLC (**A, B**) EMQA was more potent against NSCLC than FTY720. (A) The viabilities of NSCLC cells after exposure to EMQA and FTY720 at indicated doses for 72 hours were determined by MTT. Point: mean, error bar: S.D. (*n* = 6) (B) The percentage of apoptotic and necrotic cell death induced by EMQA and FTY720 at indicated doses for 48 hours were determined by Annexin-V/PI staining. Block bar: mean of apoptotic cell, gray bar: mean of necrosis cell. (*n* = 3) (**C**) The PP2A activities of NSCLC cells were determined after exposure to EMQA 5 μM and FTY720 10 μM for 24 hours. Bar, mean; error bars, S.D. (*n* = 3) (**D**) EMQA and FTY720 induced downregulation of p-Akt and apoptosis of NSCLC cells. Three different NSCLC cell lines exposed to EMQA or FTY720 at indicated doses were analyzed by western blot. Representative western blot images of three identical experiments are shown.

Given the vital role of SET suggested by above data, we're interested to know whether SET could serve as a good target for the development of future anti-cancer treatment. In the past few years, our team focused on investigating potential PP2A enhancer as anti-cancer treatment. A novel small molecule compound, EMQA (previously named TD19), was recently identified to inhibit SET-mediated PP2A inactivation. In addition, FTY720, a sphingosine analogue was reported to exert anti-tumor properties via targeting SET-PP2A binding [29]. We used three different methods to evaluate the anti-NSCLC effects of EMQA and FTY720. As shown in Figure 3A, both EMQA and FTY720 impaired the viabilities of all NSCLC cells in a dose-dependent manner, but the doses required for EMQA to achieve more than 50% inhibition on cell survival were much lower than FTY720. By Annexin V/PI double staining, we further characterized the proportion of cells underwent apoptotic and necroptotic cell death induced by EMQA and FTY720. As shown in Figure 3B, both apoptotic and necroptotic cell death contributed to the anti-NSCLC effects of EMQA and FTY720. Taken apoptotic and necroptotic death into account, the number of EMQA-induced cell death was nearly two times more than that of FTY720. Furthermore, we examined the PP2A activities of NSCLC cells after exposing to EMQA and FTY720, and found that the incremental changes of PP2A activities induced by EMQA was also higher than that of FTY720 (Figure 3C). Importantly, western blot analysis showed corresponding changes; EMQA-mediated downregulation of p-Akt and induction of PARP signaling were more potent than that of FTY720 at the same dose level (Figure 3D). Taken together, EMQA exerts better anti-NSCLC properties than FTY720 through improving PP2A-mediating p-Akt downregulation.

### SET antagonist work synergistically with paclitaxel in NSCLC cells via promoting PP2A-mediated p-Akt downregulation

**Figure 4:**
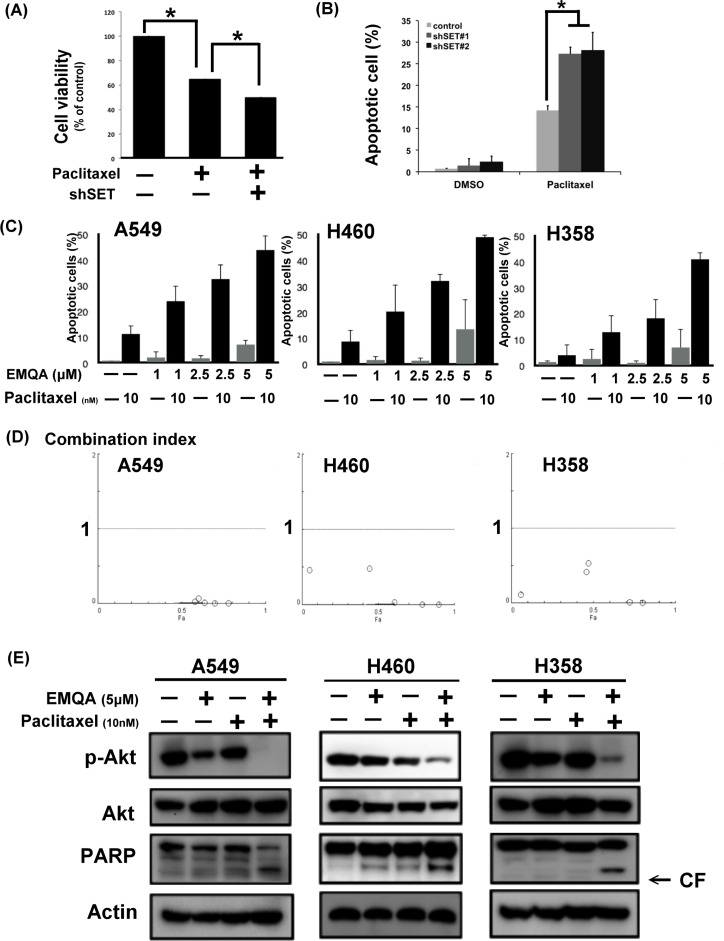
Antagonizing SET enhancing the effects of paclitaxel on NSCLC (**A, B**) Knockdown SET by shRNA significantly enhanced the pro-apoptotic effects paclitaxel. (A) The viability of A549 cells with or without SET knockdown after exposure to paclitaxel 10 nM for 48 hours. Bar: mean, error bar: S.D. (*n* = 6) (B) The percentage of apoptotic A549 cells with or without SET knockdown after exposure to paclitaxel 10 nM for 48 hours. Bar: mean, error bar: S.D. (*n* = 3) (**C**) The dose-dependent pro-apoptotic effects of paclitaxel combined with the novel SET antagonist, EMQA. Three different NSCLC cell lines exposed to the indicated treatments were analyzed by FACS (sub-G1 analysis). Bar: mean, error bar: S.D. (*n* = 3) (**D**) The combination index of three different NSCLC cell lines was determined by the results of sub-G1 analysis. (**E**) EMQA plus paclitaxel induced downregulation of p-Akt and apoptosis of NSCLC cells. Three different NSCLC cell lines exposed to EMQA 5 μM and/or paclitaxel 10 nM were analyzed by western blot. Representative western blot images of three identical experiments are shown.

**Figure 5:**
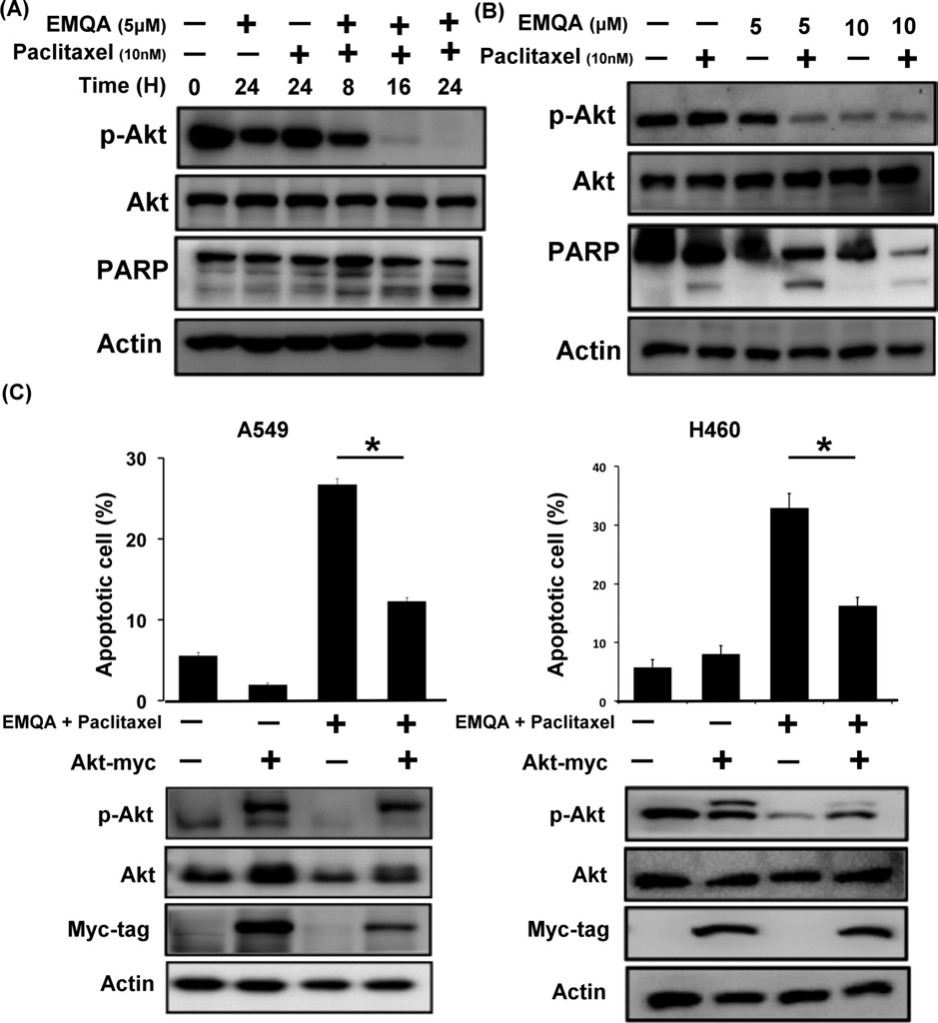
Downregulation of p-Akt determines the synergism of EMQA and paclitaxel combination treatment (**A**) The time-dependent effects of EMQA and paclitaxel combination treatment on p-Akt and poly(ADP-ribose) polymerase (PARP) in A549 cells. (**B**) The dose-dependent effects of EMQA and paclitaxel combination treatment on p-Akt and PARP in A549 cells. (**C**) Ectopic expression of Akt-myc diminished the effects of EMQA and paclitaxel combination treatment on apoptosis in A549 and H460 cells. After transfecting A549 and H460 cells with Akt-myc for 24 hours, cells were treated with EMQA 5 μM and paclitaxel 10 nM for 24 hours and analyzed by flow cytometry (sub-G1) and western blot. Bar, mean; error bars, S.D. (*n* = 3).

Since SET was shown to promote resistance to paclitaxel in our previous data, we aimed understand whether targeting SET would be a potential approach to reverse chemoresistance of NSCLC cells. As shown in Figure 4A and 4B, exposing to paclitaxel inhibited the viabilities of A549 cells, while additive SET inhibition further enhanced the cytotoxic effects of paclitaxel. Next, we assessed the therapeutic effects on combining SET antagonist to paclitaxel. Compared with paclitaxel single treatment, the numbers of apoptotic cells were significantly increased in lung cancer cells co-treated with EMQA and paclitaxel (Figure 4C). The combination index of the three NSCLC cell lines tested was below one, which indicated a synergism between the SET antagonist and paclitaxel treatment (Figure 4D). To further illustrate the molecular events that occurred upon co-treatment, we analyzed the cell lysate treated with paclitaxel 10 nM and/or EMQA 5 μM by western blot. Corresponding to prior data, we found that the expression of p-Akt was significantly decreased in all lung cancer cell lines treated with EMQA and paclitaxel (Figure 4E). Furthermore, co-treatment-induced downregulation of p-Akt was shown in a dose- and time-dependent manner (Figure 5A and 5B). Importantly, the activation of PARP signaling corresponded to downregulation of p-Akt. To further validate the role of Akt, we generated A549 and H460 cells with ectopic expression of myc-tagged Akt by transient transfection and treated them with EMQA 5 μM and paclitaxel 10 nM. As shown in Figure 5C, the efficacies of co-treatment was significantly reduced in the Akt-overexpressed cells. Together, our findings indicate that inhibition of Akt signaling determines the synergistic effects of EMQA and paclitaxel in NSCLC cells.

### Validation of the role of SET-PP2A in the anti-NSCLC mechanism of EMQA and paclitaxel co-treatment

**Figure 6:**
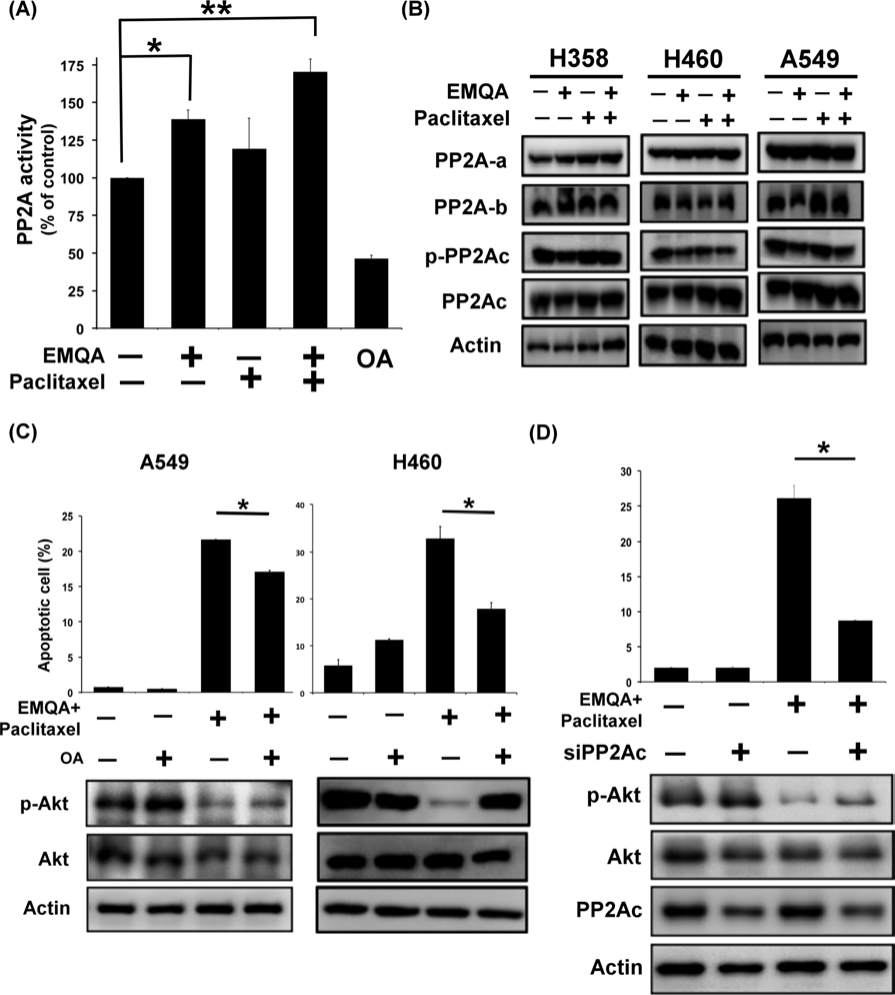
Validation of the role of PP2A in the cytotoxic effects of EMQA and paclitaxel combination treatment (**A**) The PP2A activities of A549 cells were determined after exposure to EMQA 5 μM and/or paclitaxel 10 nM for 24 hours. Bar, mean; error bars, S.D. (*n* = 3) (**B**) The effects of EMQA and/or paclitaxel on all the sub-units of PP2A complex. Representative western blot images of three identical experiments were shown. (**C**) Co-treatment with PP2A inhibitor, okadaic acid (OA), reduced the effects of EMQA and paclitaxel combination treatment on p-Akt and apoptosis. A549 and H460 cells were treated with EMQA 5 μM plus paclitaxel 10 nM and/or OA 100 nM for 24 hours and analyzed by flow cytometry and western blot. Bar, mean; error bars, S.D. (*n* = 3) Downregulating PP2Ac by siRNA diminished the pro-apoptotic effects of EMQA and paclitaxel combination treatment in A549 cells. A549 cells were first transfected with PP2Ac siRNA or mock for 24 hours and exposed to EMQA 5 μM plus paclitaxel 10 nM for 24 hours. Cell apoptosis was analyzed by flow cytometry and the associated molecular alterations were analyzed by western blot. Bar, mean; error bars, S.D. (*n* = 3).

Since EMQA targets SET, the PP2A inhibitor, and PP2A is an important negative regulator of p-Akt, we next sough to examine whether and how PP2A was affected by co-treatment of EMQA and paclitaxel. NSCLC cells were treated with EMQA 5 μM and/or paclitaxel 10 nM, and analyzed by western blot and PP2A activity assay. Notably, the PP2A activities in cells treated with EMQA and EMQA plus paclitaxel were significantly increased than control (Figure 6A), without affecting the expressions of PP2A subunits (Figure 6B). Next, we exposed A549 and H460 cells to okadaic acid (OA), a PP2A inhibitor, and/or EMQA-paclitaxel co-treatment. As shown in Figure 6C, OA treatment not only enhanced the expression of p-Akt, but also diminished the pro-apoptotic effects of EMQA-paclitaxel co-treatment in A549 and H460 cells. Correspondingly, when we used siRNA to knockdown PP2Ac specifically, the effects of EMQA and paclitaxel co-treatment were significantly reversed (Figure 6D). Taken together, our findings indicate that EMQA and paclitaxel co-treatment downregulates p-Akt by enhancing the activity of PP2A in NSCLC cells.

### EMQA reactivates PP2A by disrupting SET-mediated PP2A inactivation

**Figure 7:**
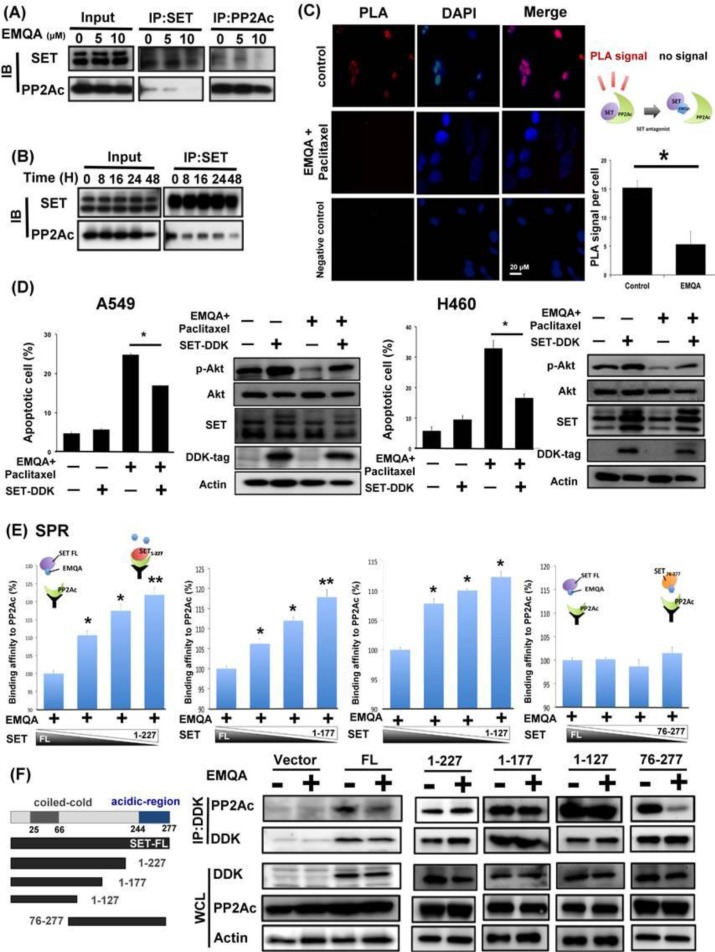
EMQA reactivates PP2A in NSCLC cells by disrupting the SET-PP2Ac binding (**A**) Does-dependent effects of EMQA on SET-PP2Ac binding. After exposure to EMQA treatment at the indicated doses, A549 cells were harvested and analyzed by co-immunoprecipitation. The expression of PP2Ac in the SET-immunoprecipitant complex (middle panel) and the expression of SET in the PP2Ac-immunoprecipitant complex (right panel) decreased with increasing dose of EMQA. (**B**) Time-dependent effects of EMQA on SET-PP2Ac binding. A549 cells were collected after exposure to 5 μM EMQA at the indicated times and analyzed by co-immunoprecipitation. Western blot analysis of the SET-immunoprecipitant complex showed that EMQA disrupted SET-PP2Ac binding in a time-dependent manner. (*n* = 3) (**C**) Proximal ligation assay (PLA) revealed that EMQA plus paclitaxel treatment significantly reduced the SET-PP2Ac binding in A549 cells. (Red dots in PLA image, nucleus stained with DAPI in blue) The corresponding quantification is shown on the right side. Bar: mean, error bar: S.D. (**D**) Ectopic expression of SET-myc diminished the effects of EMQA and paclitaxel combination treatment on inhibition of p-Akt and apoptosis induction. After transfecting A549 and H460 cells with SET-myc for 24 hours, cells were treated with EMQA 5 μM and paclitaxel 10 nM for 24 hours and analyzed by flow cytoemetry and western blot. Bar, mean; error bars, S.D. (*n* = 3) (**E**) The 227–277 sequence at the C-terminal of SET protein is critical for the effect of EMQA. Four different recombinant truncated SET proteins (as shown in Figure 7F, left panel), SET^1–127^, SET^1–177^, SET^1–227^ and SET^76–277^ were generated and mixed the full-length SET protein in the indicated proportions. Under a fixed concentration of EMQA, the binding affinities of the indicated protein mixture to PP2Ac were detected in a BIAcore T200 system. (*n* = 3) The symbolic binding relationships between SET proteins and PP2Ac were illustrated. (**F**) After transfecting A549 cells with vectors coding full-length SET-DDK, SET^1–127^-DDK, SET^1–177^-DDK, SET^1–227^-DDK and SET^76–277^-DDK or 24 hours, these cells were treated with EMQA 5 μM and analyzed by co-immunoprecipitation. Co-immunoprecipitation analysis showed that EMQA significantly decreased the SET^FL^-PP2Ac and SET^76–277^ binding, while the association of PP2Ac and other truncated SET proteins were not affected. Left panel showed the schema of the truncated SET proteins.

EMQA was found to enhance PP2A activity via targeting SET. To validate the mechanism of action, we used three different strategies to elucidate how EMQA affects SET-PP2Ac binding. As shown in Figure 7A and 7B, the expressions of PP2Ac in the SET-immunoprecipitant complex were decreased by EMQA treatment in a dose- and time-dependent manner. Correspondingly, increasing dose of EMQA also led to decreasing expression of SET in the PP2Ac immunoprecipitant complex (Figure 7A, right panel). Furthermore, we used proximal ligation assay (PLA) to demonstrate the effects of EMQA-paclitaxel co-treatment in individual cells. As shown in Figure 7C, high PLA signals were presented in the untreated A549 cells and significantly diminished in the cells exposed to EMQA and paclitaxel. Next, we overexpressed SET in A549 and H460 cells and treated them with EMQA and paclitaxel. As illustrated in Figure 7D, the effects of EMQA and paclitaxel co-treatment on promoting apoptosis and inhibiting p-Akt expression were diminished by overexpressing SET. These data validate the role of SET in mediating the effects of EMQA and paclitaxel co-treatment. To elaborate the target sites of EMQA, we generated four truncated forms of SET proteins (SET^1–127^, SET^1–177^, SET^1–227^, and SET^76–277^, Figure 7F). First, we tested the effects of EMQA on the binding affinities between PP2Ac and these truncated SET proteins (Figure 7E). Using a cell-free surface plasmon resonance (SPR) system, we exposed different proportions of full-length and truncated SET to a fixed-dose of EMQA and PP2Ac protein. Interestingly, we found that the effects of EMQA on affecting the binding affinities between full-length SET to PP2Ac and SET^76–277^ to PP2Ac were similar (Figure 7E). Conversely, with increasing proportion of SET^1–127^, SET^1–177^ and SET^1–227^ in the SET protein complex, the signals bound to PP2Ac were significantly increased. The data indicated that only the truncated SET containing its C-terminal fragment (SET^76–277^) was targeted by EMQA. To validate the SPR findings, we generated A549 cells with ectopic overexpression of DDK-tagged truncated SET protein by transient transfection. As shown in Figure 7F, only the binding of PP2Ac with full-length SET and SET^76–277^ were disrupted by EMQA.

### The *in vivo* synergistic anti-cancer effects of EMQA and paclitaxel

**Figure 8:**
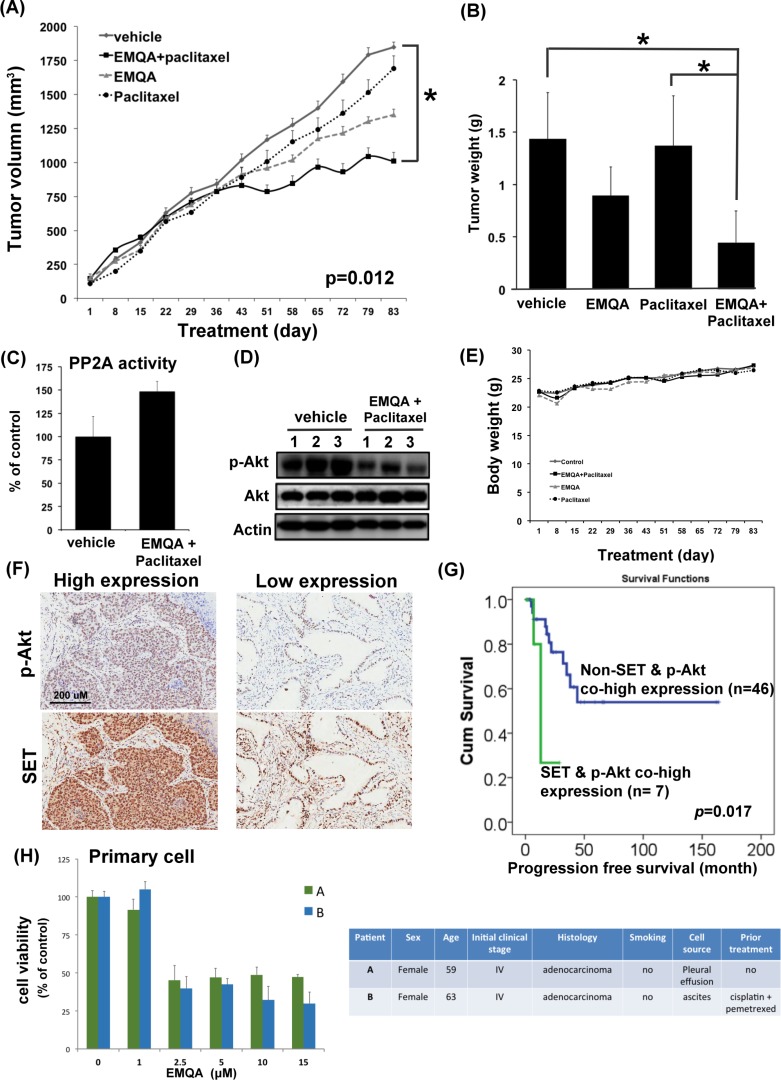
The *in vivo* effects and clinical relevance on targeting SET/PP2A/p-Akt for the treatment of NSCLC (**A**) The growth curves of A549 xenografted tumor in nude mice treated with vehicle, paclitaxel 3 mg/kg twice a week and/or EMQA 5 mg/kg/day. Points, mean; Bar, S.E. *P <0.05 (*n* = 10 in each treatment arm) (**B**) The average tumor weight of resected A549 xenografted tumor after exposing to vehicle, paclitaxel and/or EMQA treatments measured at the end of experiments. Bar, mean; error bar, S.D. (**C**) Analysis of PP2A activity of A549 xenografted tumor in vehicle- and EMQA and paclitaxel- treated nude mice. Bar, mean; error bar, S.D. (*n* = 10) (**D**) Representative western blot image of the expression of p-Akt and Akt in A549 xenografted tumor lysate. (**E**) The average body weights of nude mice exposed to the indicated treatments. (**F**) The expression level of SET and p-Akt were significantly correlated in the tumors. Representative images of the immunohistochemical staining of SET and p-Akt in tumors obtained form identical patient. (**G**) Co-high expression of SET and p-Akt is a poor prognosis factor in NSCLC patients. Progression-free survival from the time of diagnosis in NSCLC patients with and without co-expression of SET and p-Akt was compared by log-rank test. (**H**) EMQA induced cell growth inhibition on primary malignant cells obtained from two NSCLC patients. Viabilities of primary cells gathered from patient A and B after exposure to EMQA at indicated doses for 72 hours were assessed by MTT (left panel). Bar: mean, error bar: S.D. The basic characteristics of patient A and B were summarized at right panel.

To test the *in vivo* anti-tumor effects of combining EMQA and paclitaxel, we generated an A549 xenografted mouse model and treated mice with vehicle, paclitaxel 3 mg/kg twice a week and/or EMQA 5 mg/kg per day. Compared to mice receiving EMQA or paclitaxel alone, the tumor growth rate of mice receiving paclitaxel and EMQA was significantly reduced (Figure 8A). The average tumor weight of mice receiving combination treatment measured at the end of the study of was much lower than mice in other treatment arms (Figure 8B). We also analyzed the tumor lysate by western blot and PP2A activity assay. In concordance with our previous results, the PP2A activity in tumors taken from mice receiving combination therapy were significantly higher than those receiving vehicle (Figure 8C), and their expressions of p-Akt were also downregulated (Figure 8D). Notably, there was no obvious difference in the body weights of mice exposed to different treatments, which suggest that this combination regimen was tolerable to mice (Figure 8E).

### The clinical relevance of SET/PP2A/p-Akt signaling in NSCLC

To understand the clinical impacts on targeting SET/PP2A/p-Akt signaling in NSCLC patients, we examined the expression of p-Akt and explored it relationship with SET expression in tumor tissues. As shown in Figure 8F, we found that the expression of SET was significantly correlated with the expression of p-Akt (*p* = 0.031). Furthermore, we found that patients with concomitant overexpression of SET and p-Akt had significantly shorter progression-free-survival after initial diagnosis (Figure 8G). Lastly, we tested the effects of EMQA on the primary lung cancer cells obtained from two different patients with NSCLC (Figure 8H). Using MTT assay, we found that EMQA inhibited the viability of both cells with an IC_50_ below 2.5 μM.

## DISCUSSION

In the current report, we described the clinical relevance of SET expression in NSCLC patients and characterized the tumor promoting properties of SET in NSCLC cells. Furthermore, we found that SET-mediated PP2A inactivation may be responsible for the occurrence of chemoresistance in NSCLC cells, and additive knockdown of SET restored the sensitivity of NSCLC cells to paclitaxel. Moreover, we showed the anti-NSCLC efficacies of SET antagonist alone or in combination with paclitaxel *in vitro*, *in vivo* and on primary NSCLC cells. Our findings increase knowledge about the oncogenic role of SET in NSCLC, and also suggest a novel strategy through which to sensitize NSCLC to chemotherapy.

Many different mechanisms have been proposed to explain the occurrence of chemoresistance in cancer cells, such as the appearance of multidrug resistance transporters resulting in decreased intra-tumor drug concentrations, reduced drug activation by increased detoxificantion of drugs, and alterations in the drug target or apoptosis regulatory genes [30–33]. However, the above-mentioned mechanisms largely focused on the “treatment-induced” resistance and might not address other potential mechanisms of chemoresistance, particularly the cause of primary resistance. In this study, we found that SET was upregulated in the tumors obtained from NSCLC patients upon diagnosis (Figure 1A). Ectopic expression of SET diminished the anti-tumor properties of paclitaxel (Figure 2D). Transient knockdown of SET alone didn't lead to activation of apoptosis signal (Supplementary Figure 1) but silencing SET by shRNA led to promotion of paclitaxel-induced apoptosis of NSCLC cells (Figure 4A and 4B). Furthermore, we showed that the synergistic anti-cancer effects by combining a novel SET antagonist, EMQA, and paclitaxel, and the synergism of this combination was determined by inhibition SET/PP2A/p-Akt signaling (Figures 5–7). Intriguingly, upreguation of Akt signaling was reported to contribute to the chemoresistance to cisplatin and docetaxel in lung cancer cell lines, and use of LY294002, a PI3K/Akt inhibitor, showed synergy with docetaxel. [33, 34] From our current observations and previous findings, we concluded that SET-induced PP2A inactivation may play a critical role mediating the upregulation of p-Akt and contributing to the occurrence of chemoresistance in NSCLC cells. However, current data is not sufficient to fully illustrate the role of SET in mediating secondary chemoresistance. We tried to validate the potential role regarding this property by examining SET expression after exposing NSCLC cells to paclitaxel, but found no significant changes in the mRNA or protein expression of SET (Supplementary Figure 2). Further studies are needed to address this issue.

PP2A is a major serine/threonine phosphatase that regulates various cellular processes, such as signal transduction, cell cycle determination, apoptosis, protein synthesis, metabolism and stress response [29, 35, 36]. As OA, a PP2A inhibitor, promotes tumor growth, and inactivation of PP2A is required for the cellular malignant transformation induced by SV40 and RalA, the tumor suppressor role of PP2A is strongly suggested.[15, 35, 37, 38] Importantly, aberrant expression of the PP2A inhibitors, such as SET and cancerous inhibitor of PP2A (CIP2A), in tumor cells are the major causes of PP2A dysfunction in human cancers. [12, 25, 39] For NSCLC, the oncogenic role of SET has less been discussed till recently. The study published by Liu et al. was the first to show that 91.4% of NSCLC tumors were positive for SET expression, and high SET expression was associated with advanced clinical stage and worse survival of patients [16]. The authors also found that SET knockdown impaired the proliferative and invasive potential of NSCLC cells through enhancing PP2A-mediated inhibition of oncogenic signals, including AKT. In this report, we validated the high prevalence and tumor-specificity of SET expression in NSCLC (Figure 1A), and demonstrated the closely correlated expressions of SET and p-Akt (Figure 8F). Furthermore, we found that co-high expression of SET and p-Akt defined a specific subgroup of patients with rapid progression potential (Figure 8G). Taken together, the results of Liu's and our study demonstrated the importance of SET/PP2A/p-Akt signal in NSCLC patients, and justified the rationale of targeting SET for the treatment of NSCLC.

By far, FTY720 and OP449 were the most frequently studied SET antagonists. [11, 14, 15, 29] FTY720 was originally designed and developed as an immunosuppressant to treat patients with relapsing multiple sclerosis [40]. Interestingly, subsequent studies showed that FTY720 exerts an “off-target” effect activating PP2A by dissociating SET-PP2Ac interactions. [11, 25, 29] On the other hand, the apolipoprotein-E mimetic peptide, OP449 (previously named COG112), was specifically designed to bind to the SET protein and antagonizes the SET-mediated PP2A inactivation.[24, 26] These two compounds have been tested in several different types of cancers with promising results [11, 24–26, 29]. In this study, we introduced the efficacy and detail mechanism of action of a novel compound, EMQA, and compared it with FTY720 for the treatment of NSCLC. Interestingly, we showed that EMQA and FTY720 both were active compounds against NSCLC, while EMQA exert more potent anti-tumor properties (Figure 3). As we detailed in material section, this compound was originally developed to enhance PP2A via targeting another PP2A inhibitor, CIP2A [41], but whether the additive CIP2A suppression contributed to better anti-tumor effects of EMQA was not clear. More studies are needed to clarify this observation. Collectively, our findings and previous reports suggest that reactivating PP2A by targeting SET may be a feasible and highly potential direction to develop novel treatment against NSCLC and other malignant disease [12, 24, 25, 39, 42].

In order to detail the mechanisms by which the small molecular compound, EMQA, affects the binding between SET and PP2Ac, we generated four different truncated SET proteins and tested them using SPR (Figure 7E) and co-immunoprecipitation (Figure 7F). Distinct from the full-length and the truncated SET_76–277_ proteins, we found EMQA could not affect the binding between the PP2Ac and truncated SET proteins without the C-terminal. Our data indicated that the Asp/Glu-rich C-terminus of SET protein is required for the EMQA drug effects. [43] Notably, both OP449 and FTY720 were found to target the last 100 amino acid in the C-terminus, almost identical to EMQA. [18, 23, 24] These findings support our previous notions that the Asp/Glu-rich acidic region serves as the protein domains involved in protein-protein interaction and transcriptional activation [44–46].

## CONCLUSION

In this study, we characterized the role of the oncoprotein SET in NSCLC. We found that SET protein is critically involved in the carcinogenesis and the development of chemoresistance of NSCLC. More importantly, we showed that a novel SET antagonist, EMQA, reactivates PP2A and sensitizes NSCLC cells to paclitaxel treatment *in vitro* and *in vivo*. Our results not only illustrate a potential mechanism of chemoresistance, but also provide evidence to support further development of SET antagonists for the treatment of NSCLC.

## MATERIALS AND METHODS

### Reagents and antibodies

Paclitaxel was kindly provided by Bristol-Myers Squibb Pharmaceuticals (NY, USA), FTY720 was provided by Norvatis (Basel, Switzerland) and okadaic acid (OA) was purchased from Cayman Chemical (Ann Arbor, MI). EMQA (N^4^-(3-Ethynylphenyl)-6, 7-dimethoxy-N^2^-(4-phenoxyphenyl) quinazoline-2, 4-diamine), previously named TD-19 [41], was a novel PP2A enhancer designed and developed based on the quinazoline backbone as novel PP2A enhancer. The original hypothetical mechanism of EMQA to reactivate PP2A was through inhibition of CIP2A and the property as SET antagonist was identified and reported here. For *in vitro* studies, drugs were dissolved in dimethyl sufoxide (DMSO) at various concentrations and added to cells in RPMI 1640 medium containing 5% fetal bovine serum (FBS). The final DMSO concentration was 0.1% after adding to the medium. Antibodies for immunoblotting including anti-PP2Ac, -Akt, and -PARP were purchased from Santa Cruz Biotechnology (San Diego, CA), anti-DDK-Tag was obtained from Origene (Rockville, MD) and anti-SET was obtained from Bethyl (Montgomery, TX). Other antibodies including anti-p-Akt and -Myc-tag were purchased from Cell Signal (Danvers, MA).

### Cell culture and western blot analysis

The A549, H460 and H358 cell lines were obtained from the Bioresource Collection and Research Center (BCRC; Hsinchu, Taiwan). Cells were maintained in RPMI 1640 medium with 10% FBS in a 37°C humidified incubator in an atmosphere of 5% CO_2_ in air. After exposure to treatments at the indicated concentrations and time periods, cell lysates were prepared for immunoblotting and co-immunoprecipitation (co-IP). The procedure of co-IP and western blot analysis were performed as previously described [47].

### Cell viability and apoptosis analysis

The viabilities of NSCLC cells with and without SET knockdown were determined by MTT assay. The numbers of apoptotic cells were determined by flow cytometry (sub-G1) and western blot analylsis of PARP. Annexin-V/PI double staining assay was used to determine the numbers of both apoptosis and necrotic cells. Synergism of paclitaxel and EMQA were determined by the software package CalcuSyn (Biosoft, Cambridge, UK) and a combination index less than 1 was defined as synergism.

### Colony formation and sphere formation assay

For colony formation assay, A549 cells with and without SET knockdown were seeded in 10 cm plates at a density of 1000 cells per well and culture for 14 days. Colonies were fixed with 3.75% of paraformaldehyde solution, stained with 0.5% crystal violet, and analyzed by inverted microscope. For the generation of tumor spheres, 10,000 cells were plated in 6-well ultra-low attachment plates (Corning) and grown in serum-free medium. After 7 days, plates were analyzed for sphere formation.

### Gene knockdown using siRNA and shRNA

SMARTpool small interfering RNA (siRNA) reagents, including control (D-001810–10) and PP2A-C (L-003598–01) were purchased from Dharmacon (GE; Lafayette, CO). The SET short hairpin RNA (shSET) were obtained from National RNAI Core Facility Platform (Taiwan). In brief, cells were first transfected with siRNA (final concentration, 100 nM) using the Dharma-FECT4 transfection reagent (Dharmacon) according to the manufacturer's instructions for 48 hours. The efficacy of genetic knockdown was confirmed by western blot.

### Transient transfection

The cDNAs of SET and Akt were obtained from Origene (Rockville, MD, USA), and cDNAs of truncated SET (1–77, 1–127, 1–227 and 76–277) were generated in our lab. Following transfection for 48 hours, cells were exposed to EMQA for 24 hours and subsequently harvested for further analysis.

### PP2A phosphatase activity

The protein phosphatase activity in each cell lysate was determined by measuring the generation of free phosphate from threonine phosphopeptide using the malachite green–phosphate complex assay as described by the manufacturer (Upstate Biotechnology, Lake Placid, NY, USA). In brief, PP2A-specific reaction butter (Milipore, Billerica, MA, USA) containing 750 mM phosphopeptide substrate was added into cell lysates prepared in a low-detergent lysis buffer. After incubation for 10 min at 30°C, the malachite dye was added and free phosphate was measured by optical density at 650 nm.

### Quantification of SET gene expression

The endogenous SET mRNA expression of NSCLC cells were assessed by qPCR. According to the manufacturer's instructions, total RNA from the cell was extracted from indicated cell lines using PureLink RNA mini kit (ThermoFisher) and than reversely transcribed using a SuperScript VILO Master Mix (Invitrogen). The real-time quantitative polymerase chain reaction (PCR) was performed on a 7500 Real-Time PCR System (Applied Biosystem) with a specific primer set of target gene and SYBR Green dye for detection. GAPDH was used as the reference gene and experiments were repeated in triplicate. The relative level of gene expression of indicated cell lines to A549 was calculated by the 2^−ΔΔCt^ method.

Gene-specific primer pairs used in this experiment are:

SET
Forward: 5′-GCTCAACTCCAACCACGAC3′
Reverse: 5′-TCCTCACTGGCTTGTTCATTA3′
GAPDH
Forward: 5′-GGAAGGTGAAGGTCGGAGT3′
Reverse: 5′-TGAGGTCAATGAAAGGGGTC3′

### Proximal Ligation assay (PLA)

A549 cells were seeded on glass cover slides overnight and subsequently exposed to co-treatment of EMQA and paclitaxel or DMSO for 24 hours. The *in vivo* binding of SET and PP2Ac was assessed using the Duolink II Detection Kit (Sigma). The primary probe antibodies were monoclonal mouse anti-PP2A and rabbit anti-SET antibodies.

### Expression of recombinant protein

The plasmid-containing human full-length SET was obtained from OriGene (RC207606, Rockville, MD). The cDNA of full-length and four truncated forms of SET (1–127, 1–177, 1–227 and 76–277) were subcloned into the BamH I and Not I sites of the pCMV6-Entry vector. The FLAG fusion protein of FL and truncated mutations of SET were expressed in *Escherichia coli* and affinity purified using HisTALON gravity columns (Takara Clontech). PP2Ac-GST was purchased from Abnova (H00005515, Abnova, Taiwan) and SET-His recombinant proteins used for SPR were obtained from Genway (GWB-ATG319, GenWay, San Diego).

### Surface plasmon resonance (SPR)

Binding affinities of full-length SET and truncated SET to PP2Ac and the effect of EMQA on disrupting SET and PP2Ac were analyzed on a BIAcore T200 biosensor system (GE). In brief, PP2Ac-GST recombinant protein was bound in the CM5 chip pre-coated with GST-capture antibodies. Sensorgrams were generated by injecting several concentrations of EMQA mixed with fixed-concentration of SET recombinant protein, or the mixture of different concentration of truncated SET proteins in a fixed-dose EMQA. PP2Ac-GST recombinant protein used for this experiment was purchased from Abnova (H00005515) and SET-His recombinant protein was obtained from Genway (GWB-ATG319).

### Xenograft tumor growth

Male NCr athymic nude mice (5–7 weeks of age) were obtained from the National Laboratory Animal Center (Taipei, Taiwan). All experimental procedures using these mice were performed in accordance with protocols approved by the Institutional Laboratory Animal Care and Use Committee of National Taiwan University. Each mouse was inoculated s.c. in the dorsal flank with 1 × 10^6^ A549 cells suspended in 0.1 ml of serum-free medium containing 50% Matrigel (BD Biosciences, Bedford, MA). Mice with successful inoculation of A549 xenografted tumors were treated with vehicle, paclitaxel 3 mg/kg/BIW by intra-peritoneal injection, and/or EMQA 5 mg/kg/day daily by oral gavage for 12 weeks. The tumor sizes and weight of mice were measured every other day during treatments, and net weights of resected tumors were measured at the end of study. PP2A activity and expression of p-Akt of tumor lysates were determined.

### Patient samples, primary culture and immunohistochemical staining

This part of the study was approved by the ethics committee of the Institutional Review Board of Cardinal Tien Hospital, New Taipei City, Taiwan. All informed consents from sample donors were in accordance with the Declaration of Helsinki and were obtained at the time of donation.

Primary tumor tissues and cell were obtained from NSCLC patients. Patient's important clinical characteristics were collected by chart review. For IHC study, representative tissues of the lung tumor and adjacent normal tissues (if available) were carefully collected from each patient and made into a tissue array. These paraffin-embedded tumor tissue sections (4 μm) on poly-L-lysine-coated slides were deparaffinized and rinsed with 10 mM Tris-HCl (pH 7.4) and 150 mM sodium chloride before performing IHC studies. Peroxidase was quenched with methanol and 3% hydrogen peroxide. Slides were then placed in 10 mM citrate buffer (pH 6.0) at 100°C for 20 minutes in a pressurized heating chamber. After incubation with 1:500 dilution of SET antibody (A302–2621, Bethyl, Taxas) and 1:200 dilution of p-AKT antibody (SC-16646-R, Santa Cruz, San Diego, CA) for 1 hour at room temperature, slides were thoroughly washed three times with phosphate buffered saline. Bound antibodies were detected using the EnVision Detection Systems Peroxidase/DAB, Rabbit/Mouse kit (Dako, Glostrup, Denmark). The slides were then counterstained with hematoxylin. Each experiment included, for each tissue array slide, phosphate-buffered solution used as the primary antibody for the negative controls, whereas samples known to express SET or p-Akt strongly served as the positive control. Every slides was reviewed by a specific pathologist, and both the strength of staining and the percentage of positive cells were used for the calculation of H score. Tumor with high SET expression was defined as those with H score more than 190, while the H score for high p-Akt was 170 or more. For primary cell culture, fresh collected body fluid were carefully centrifuged, washed and treated with red blood cell lysis buffer for about 10 minutes. Harvested cells were subsequently cultured and maintained in RPMI culture median.

### Statistical analysis

The experimental results were reported as mean ± standard error of the mean. Comparisons of mean values were performed using the independent samples *t* test. For the clinical part, descriptive statistical analysis was used to compare baseline characteristics among patients with high and low SET expressions. Progression-free survival (PFS) was defined by the time of initial diagnosis of NSCLC to the date confirmed local recurrence or distant metastasis. The Kaplan-Meier method was used to estimate the PFS, and log-rank test was used for comparisons. Differences were considered significant at *P* value less than 0.05. All statistical analyses were performed using SPSS software for Windows (17.0 version, SPSS, Chicago, IL, USA).

## SUPPLEMENTARY MATERIAL FIGURES


