# Supplementary material for: SET antagonist enhances the chemosensitivity of non-small cell lung cancer cells by reactivating protein phosphatase 2A
Source: Oncotarget. 2015 Nov 13;7(1):638–55. doi: 10.18632/oncotarget.6313 (PMC4808023; doi:10.18632/oncotarget.6313)
Supplement: Supplementary file 1 [file oncotarget-07-0638-s001.pdf]

SET antagonist enhances the chemosensitivity of non-small cell lung cancer cells by reactivating protein phosphatase 2A

Supplementary Materials

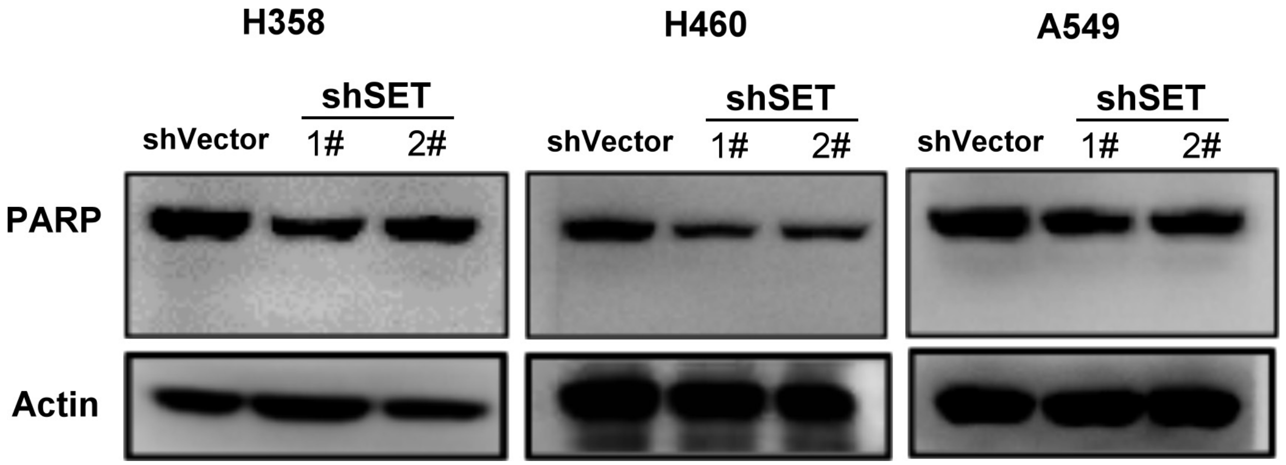

Supplementary Figure S1: Transient knockdown of SET in NSCLC cells did not lead to significant induction of apoptosis-related signal NSCLC cells with and without transient knockdown of SET were analyzed for apoptosis related signal. Representative images from triplicate experiments of western blot for actin and PARP expression were shown here.

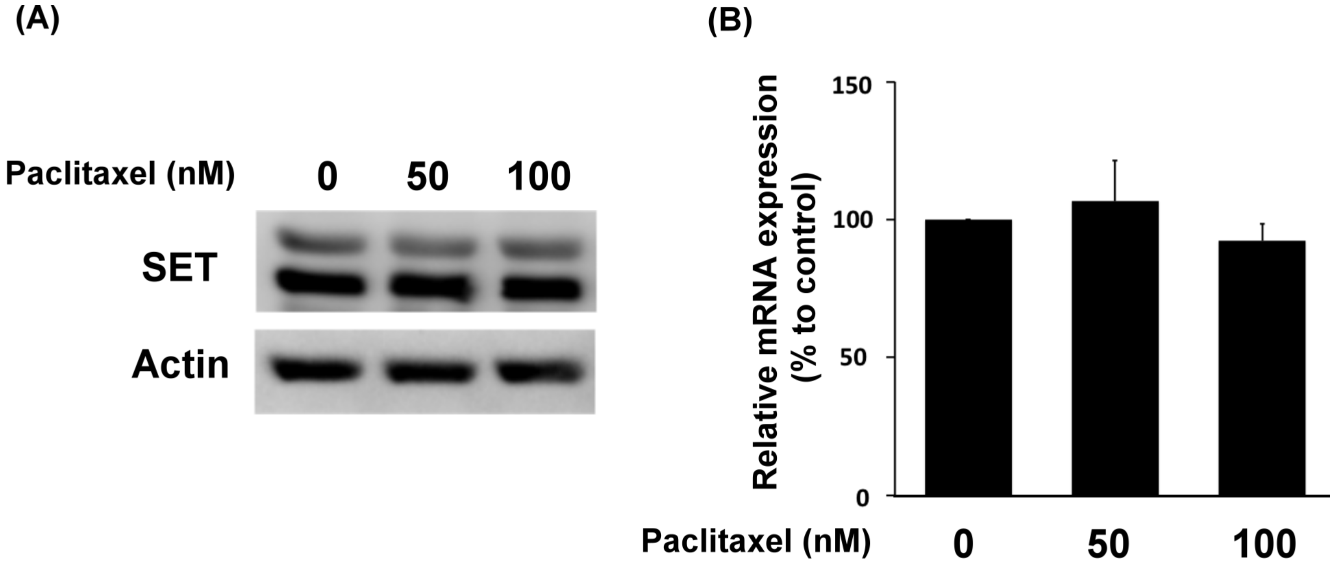

Supplementary Figure S2: Short-term paclitaxel treatment did not affect SET expression in NSCLC cells. A549 cells were exposed to paclitaxel at indicated doses for 24 hours and collected for western blot (**A**) and RT-PCR analysis (**B**). Bar: mean; Error bar: S.D.
